# ICU Admission Levels of Endothelial Biomarkers as Predictors of Mortality in Critically Ill COVID-19 Patients

**DOI:** 10.3390/cells10010186

**Published:** 2021-01-19

**Authors:** Alice G. Vassiliou, Chrysi Keskinidou, Edison Jahaj, Parisis Gallos, Ioanna Dimopoulou, Anastasia Kotanidou, Stylianos E. Orfanos

**Affiliations:** 1 First Department of Critical Care Medicine & Pulmonary Services, GP Livanos and M Simou Laboratories, School of Medicine, National and Kapodistrian University of Athens, Evangelismos Hospital, 10675 Athens, Greece; alvass75@gmail.com (A.G.V.); chrysakes29@gmail.com (C.K.); idimo@otenet.gr (I.D.); akotanid@med.uoa.gr (A.K.); 2 First Department of Critical Care Medicine & Pulmonary Services, School of Medicine, National and Kapodistrian University of Athens, Evangelismos Hospital, 10676 Athens, Greece; edison.jahaj@gmail.com; 3 Health Informatics Laboratory, School of Health Sciences, National and Kapodistrian University of Athens, 11527 Athens, Greece; parisgallos@yahoo.com; 4 Second Department of Critical Care, School of Medicine, National and Kapodistrian University of Athens, Attikon Hospital, 12462 Athens, Greece

**Keywords:** endotheliopathy, COVID-19, sE-selectin, sP-selectin, angiopoietin, sICAM-1, mortality

## Abstract

Endotheliopathy is suggested to be an important feature of COVID-19 in hospitalized patients. To determine whether endotheliopathy is involved in COVID-19-associated mortality, markers of endothelial damage were assessed in critically ill COVID-19 patients upon intensive care unit (ICU) admission. Thirty-eight critically ill COVID-19 patients were included in this observational study, 10 of whom died in the ICU. Endothelial biomarkers, including soluble (s)E-selectin, sP-selectin, angiopoietin 1 and 2 (Ang-1 and Ang-2, respectively), soluble intercellular adhesion molecule 1 (sICAM-1), vascular endothelial growth factor (VEGF), soluble vascular endothelial (VE)-cadherin, and von Willebrand factor (vWf), were measured upon ICU admission. The ICU cohort was subsequently divided into survivors and non-survivors; Kaplan–Meier analysis was used to explore associations between biomarkers and survival, while receiver operating characteristic (ROC) curves were generated to determine their potential prognostic value. sE-selectin, sP-selectin, Ang-2, and sICAM-1 were significantly elevated in ICU non-survivors compared to survivors, and also associated with a higher mortality probability in the Kaplan–Meier analysis. The prognostic values of sE-selectin, Ang-2, and sICAM-1 from the generated ROC curves were greater than 0.85. Hence, we conclude that in our cohort, ICU non-survivors had higher levels of specific endothelial markers compared to survivors. Elevated levels of these markers upon ICU admission could possibly predict mortality in COVID-19.

## 1. Introduction

Lung injury in COVID-19 is facilitated by cytokine-driven vascular leaks in the lung alveolar-endothelial interface; it seems possible that the virus gains access into systemic circulation by passing from the respiratory epithelium to the endothelium [1], turning the lung into a key organ target. Patients with COVID-19 exhibit problems in multiple organs. Encephalopathy, respiratory failure, myocarditis, myocardial infarction, acute kidney injury, and a hypercoagulable state are highly prevalent in COVID-19, all of which may be influenced by altered endothelial function [1,2]. Indeed, the disease course is worse in individuals with pre-existing comorbidities that involve endothelial dysfunction, such as diabetes mellitus, hypertension, and cardiovascular disease [3]. Severe cases of COVID-19 are characterized by hyper-inflammatory and thrombotic episodes, suggesting that one of the major targets of this disease is the endothelium, one of the body’s largest organs [4]. However, it is still largely unknown whether the vascular complications seen in COVID-19 patients are due to endothelial damage. Recently, it was suggested that endotheliopathy and platelet activation are important features of COVID-19 in hospitalized patients, and are likely to be associated with critical illness and worse outcomes [5]. A postmortem evaluation of peripheral lungs from COVID-19 patients revealed diffuse alveolar damage, severe endothelial injury, and widespread thrombosis with microangiopathy [6], while an increasing number of reports discussing the role of platelet activation and aggregation in patients with severe COVID-19 is being published [7,8]. Molecular techniques, including transcriptomics, have been able to demonstrate platelet hyperactivity [9,10].

Hence, the aim of this study was to determine the role of endothelium-related molecules, which have been previously investigated as potential biomarkers for the early diagnosis and/or prognosis of sepsis and acute respiratory distress syndrome (ARDS), in the prognosis of poor clinical outcomes in the context of COVID-19. We evaluated whether elevated levels of circulating endothelial biomarkers in critically ill COVID-19 patients upon intensive care unit (ICU) admission may predict mortality.

## 2. Materials and Methods

The study was approved by the Evangelismos Hospital Research Ethics Committee (129/19-03-2020), and all procedures carried out on patients were in compliance with the Helsinki Declaration. Informed written consent was obtained from all patients or patients’ next-of-kin prior to any study procedure.

This observational, single-center study included consecutive COVID-19 patients admitted into the intensive care unit (ICU), who had not received dexamethasone, from 22 March 2020, to 25 October 2020. SARS-CoV-2 infection was diagnosed by real-time reverse transcription PCR (RT-PCR) in nasopharyngeal swabs. Following study enrolment, demographic characteristics, comorbidities, symptoms, vital signs, laboratory findings, and COVID-19-targeted compounds were recorded. Acute physiology and chronic health evaluation (APACHE II) and sequential organ failure assessment (SOFA) score were calculated upon ICU admission. Acute respiratory distress syndrome (ARDS) was assessed according to the Berlin definition [11]. Outcome was defined as the overall ICU mortality. An additional group of 17 consecutive patients admitted to the specialized COVID-19 ward (non-ICU) of our hospital was also used for comparison reasons.

Three milliliters (3 mL) of venous blood were collected within the first 24 h post ICU admission. Blood samples were collected in Vacutainer tubes containing 0.129 M (3.8%) trisodium citrate for the collection of plasma. Plasma was collected, portioned into 0.5 mL aliquots, and stored at −80 °C until it was used.

Soluble (s)E-selectin, sP-selectin, angiopoietin 1 and 2 (Ang-1 and Ang-2, respectively), soluble intercellular adhesion molecule 1 (sICAM-1), vascular endothelial growth factor (VEGF), soluble vascular endothelial (VE)-cadherin, and von Willebrand factor (vWf) were measured in plasma samples by enzyme-linked immunosorbent assay (ELISA) according to the manufacturers’ instructions (R&D Systems Inc., Minneapolis, MN, USA). The assays use two different polyclonal antibodies against the molecules as catching and tagging antibody. The researcher who performed the measurements was blinded to the samples measured.

Data are presented as mean ± standard deviation (SD) for normally distributed variables, or as median with inter-quartile range (Q1–Q3) for skewed data. The two-group comparisons were performed by the t-test or the non-parametric Mann–Whitney test, as appropriate. The chi-square test was used to examine associations between nominal variables. Correlations were performed by Spearman’s correlation coefficient. The Kaplan–Meier method was used for survival probability estimation, and the log-rank test for a two-group comparison. Receiver operating characteristic (ROC) curves were plotted thereafter, using ICU mortality as the classification variable and biomarker levels upon ICU admission as the prognostic variable, to determine the best cut-off point for each of the endothelial markers. The optimal cut-off value for predicting mortality was calculated as the point with the greatest combined sensitivity and specificity. To further examine the effect of the biomarkers’ levels that differ between survivors and non-survivors on ICU mortality in COVID-19 patients, a theoretical score was calculated based on the ROC cut-off values. Patients who had values under the cut-off received 0 points for each biomarker, and those who had values higher than the cut-off value received 1 point for each biomarker. After scoring, we used the sum of the five biomarkers of each patient to calculate the theoretical score. The minimum score for a patient is zero (0) points and the maximum is five (5) points. Minimum score signifies that the patient had low levels (under the cut-off value) on the five biomarkers and maximum score signifies that the patient had high levels on the five biomarkers. All the tests were conducted using a Type I error, α = 0.05 and Type ΙI error β = 0.20 (80% power). The analyses were performed with IBM SPSS statistical package, version 22.0 (IBM Software Group, New York, NY, USA), and GraphPad Prism, version 8.0 (GraphPad Software, San Diego, CA, USA). All the *p*-values were calculated after two-sided tests; *p*-values < 0.05 were considered significant.

## 3. Results

### 3.1. Characteristics of the Study Population

Thirty-eight adult patients were included in the final study cohort. Once all the patients had an outcome (discharge from ICU or death), they were subsequently grouped according to ICU mortality: survivors (N = 28) and non-survivors (N = 10). Laboratory measurements upon ICU admission are presented in Table 1.

The mean patient age in our ICU cohort was 63 ± 11 years. The mean admission APACHE II score was 15 ± 5 and the mean admission SOFA score was 7 ± 3. Thirty subjects (79%) were mechanically ventilated. Thirty-five of the 38 (92%) had ARDS; 3 patients had severe, 16 had moderate, and 16 patients had mild ARDS [11]. In three patients, PaO_2_/FiO_2_ was over 300 mmHg, despite the presence of diffuse infiltrates in the chest X-rays. The overall ICU mortality rate amongst our enrolled patients was 26%. The demographics, patient characteristics, endothelial biomarkers, and important outcomes of the two groups are given in Table 2.

Hypertension, hyperlipidemia, and diabetes were the most common comorbidities; non-survivors had a higher SOFA score upon ICU admission (*p* < 0.05). COVID-19-targeted treatment did not differ between survivors and non-survivors.

### 3.2. Baseline Levels of Soluble Endothelium-Related Biomarkers

Human sP-selectin, sE-selectin, angiopoietin-1, angiopoietin-2, VE-cadherin, VEGF, sICAM-1, and vWf were quantified concurrently in all the samples using dedicated ELISA assays.

**Figure 1:**
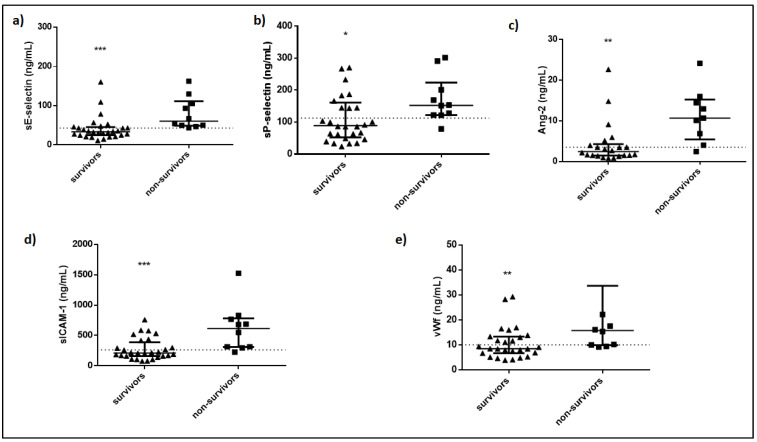
Intensive care unit (ICU) admission levels of sE-selectin, sP-selectin, Ang-2, sICAM-1, and vWf in survivors and non-survivors. (**a**) sE-selectin, (**b**) sP-selectin, (**c**) Ang-2, (**d**) sICAM-1, and (**e**) vWf levels were measured in 38 critically ill patients upon ICU admission (within 24 h). Patients were subsequently categorized as survivors (N = 28) and non-survivors (N = 10). Two-group comparisons were performed with the non-parametric Mann–Whitney test, * *p* < 0.05, ** *p* < 0.01, *** *p* < 0.001. Data are presented as scatter plots, indicating the median value and 25th to 75th centiles. Dashed line, median value of whole cohort (N = 38). Ang-2 = Angiopoietin-2; s = soluble; sICAM-1 = soluble Intercellular adhesion molecule 1; vWf = von Willebrand factor.

The biomarkers were measured in all 38 patients. At the time of ICU admission, sP-selectin, sE-selectin, Ang-2, sICAM-1, and vWf were significantly elevated in non-survivors compared to survivors (Table 2 and Figure 1).

**Figure 2:**
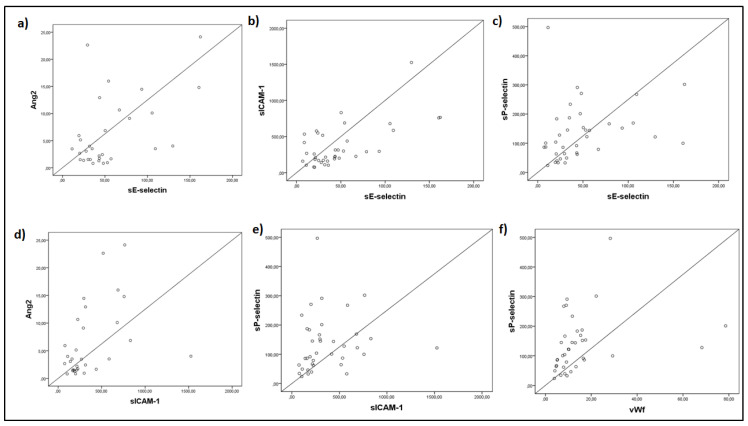
Spearman’s correlation coefficient analysis was performed on sE-selectin, sP-selectin, Ang-2, sICAM-1, and vWf levels measured upon intensive care unit (ICU) admission (within 24 h). (**a**) sE-selectin and Ang-2 (r_s_ = 0.389, *p* = 0.031), (**b**) sE-selectin and sICAM-1 (r_s_ = 0.686, *p* < 0.0001), (**c**) sE-selectin and sP-selectin (r_s_ = 0.398, *p* = 0.013), (**d**) Ang-2 and sICAM-1 (r_s_ = 0.545, *p* = 0.002), (**e**) sP-selectin and sICAM-1 (r_s_ = 0.362, *p* = 0.026), and (**f**) sP-selectin and vWf (r_s_ = 0.513, *p* = 0.001). Ang-2 = Angiopoietin-2; s = soluble; sICAM-1 = soluble intercellular adhesion molecule 1; vWf = von Willebrand factor.

Spearman’s correlation coefficient also showed that the endothelial markers exhibited strong significant correlations. A graphical representation of these correlations is presented in Figure 2.

With regard to other COVID-19 markers, sICAM-1 correlated with fibrinogen (r_s_ = 0.374, *p* = 0.02), while sE-selectin correlated with lactate dehydrogenase (r_s_ = 0.333, *p* = 0.04) and D-dimers (r_s_ = 0.374, *p* = 0.03).

**Figure 3:**
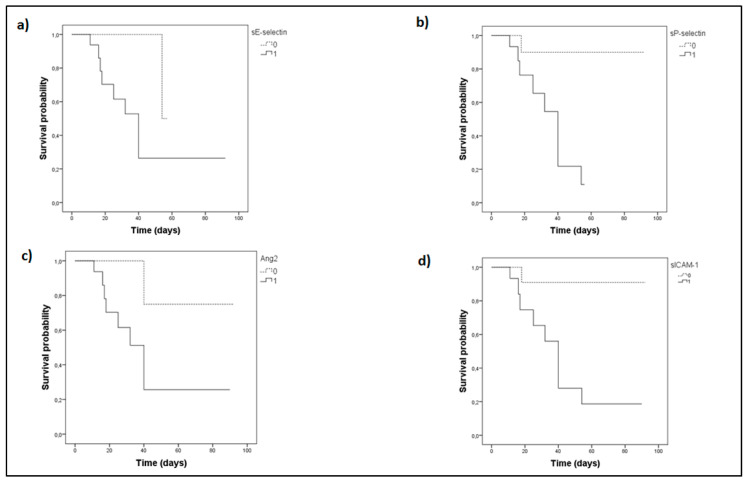
Biomarker levels on admission and intensive care unit (ICU) survival probability. (**a**) sE-selectin, (**b**) sP-selectin, (**c**) Ang-2, (**d**) sICAM-1. sE-selectin, sP-selectin, Ang-2, and sICAM-1 were measured upon ICU admission (within 24-h). The Kaplan–Meier method was used for ICU survival probability estimation and the log-rank test for two-group comparison. The ICU patient cohort was dichotomized above and below the respective median value of each biomarker. Solid lines: ≥median value (high group); dashed lines: <median value (low group). The respective median time to mortality for the two aforementioned groups were as follows: (**a**) sE-selectin, 44 days (95% CI: 28–61) for the high group, and 56 days (95% CI: 53–58) for the low group (Log-Rank test, *p* = 0.038); (**b**) sP-selectin, 35 days (95% CI: 26–43) for the high group, and 85 days (95% CI: 71–98) for the low group (Log-Rank test, *p* = 0.009); (**c**) Ang-2, 43 days (95% CI: 26–61) for the high group, and 79 days (95% CI: 57–101) for the low group (Log-Rank test, *p* = 0.048); (**d**) sICAM-1, 42 days (95% CI: 27–57) for the high group, and 85 days (95% CI: 73–98) for the low group (Log-Rank test, *p* = 0.022). Ang-2 = Angiopoietin-2; s = soluble; sICAM-1 = soluble Intercellular adhesion molecule 1.

The Kaplan–Meier method was next used for survival probability estimation. The ICU cohort was independently dichotomized above (high group) and below (low group) the medians of sE-selectin, sP-selectin, Ang-2, sICAM-1, or vWf, respectively, as given in Table 2. The probability of mortality with time was significantly elevated in the high groups (Figure 3); for vWf it tended towards but did not reach statistical significance (*p* = 0.06).

**Figure 4:**
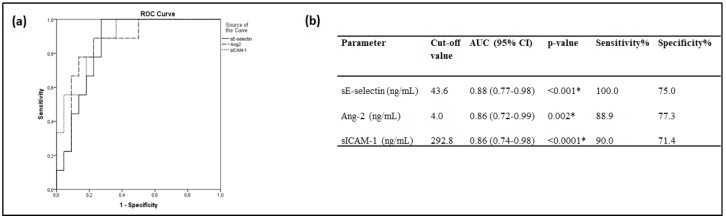
Admission biomarker levels and intensive care unit (ICU) mortality. Receiver operating characteristic (ROC) curve analysis. ROC curves were generated to determine the prognostic accuracy of either sE-selectin, Ang-2 or sICAM-1, as measured on ICU admission (within 24-h). (**a**) ROC curves; sE-selectin, solid line; Ang-2, dashed line; sICAM-1, dotted line. (**b**) The corresponding areas under the curve (AUC), 95% confidence intervals (CI) and the optimal cut-off values with the greatest combined sensitivity and specificity are given. * *p*-value < 0.05. Ang-2 = Angiopoietin-2; s = soluble; sICAM-1 = soluble Intercellular adhesion molecule 1.

ROC curves were finally generated to determine the prognostic accuracy of these endothelial markers in our cohort. sE-selectin, Ang-2, and sICAM-1 exhibited a good discriminating ability (>0.85) that was better than the predictive ability that the SOFA ROC curve exhibited in our sample; the area under the curve (AUC), the optimal cut-off points, and their sensitivities and specificities are given in Figure 4. Pair-wise comparisons of the ROC curves revealed that the three endothelial biomarkers had an equal accuracy in predicting ICU mortality (*p* > 0.05). sP-selectin and vWf, on the other hand, did not show such a good discriminating ability (0.70 < AUC < 0.80).

When we assessed the biomarkers’ theoretical calculated score in relation to ICU mortality, we found it to be significant (*p* < 0.0001) among ICU COVID-19 patients. In more detail, the theoretical mean score for survivors was 1.4 ± 1.3. On the other hand, non-survivors had a mean score of 4.1 ± 1.0.

Ang-2 and sE-selectin have been shown to be elevated in critically ill COVID-19 patients; moreover, angiopoietin-2 was suggested as a biomarker to predict transfer to the ICU [12]. Hence, we also compared the levels of all the aforementioned biomarkers on admission in ICU patients and patients admitted to the specialized COVID-19 ward of our hospital. Supplementary Table S1 shows the laboratory data of the ICU and non-ICU patients, whereas supplementary Table S2 lists demographics, patient characteristics on hospital admission, and important outcomes. Compared to ICU patients, non-ICU patients were younger, had a lower SOFA score, while 2 (12%) had mild and 2 (12%) moderate ARDS. With respect to the endothelial biomarkers measured, Ang-2 in our cohort could also discriminate between ICU and non-ICU patients; sICAM-1 and vWf were also higher, whereas sE-selectin tended to be higher in ICU patients.

## 4. Discussion

To the best of our knowledge, this is the first study that examines patterns of multiple endothelium-related indices in critically ill COVID-19 patients on ICU admission, in an effort to predict mortality. Our results indicate that in our cohort, ICU admission levels of soluble E-selectin, sP-selectin, Ang-2, sICAM-1, and vWf are higher in COVID-19 critically ill patients who will not survive. Of note, non-survivors had a cumulative theoretical predictive score, based on the aforementioned five biomarkers, of 4.1 compared to 1.4 in survivors.

Up to now, COVID-19-associated mortality has been shown to be correlated with older age, APACHE II and SOFA scores, mechanical ventilation, vasopressors, renal replacement therapy, and underlying comorbidities [13,14,15]. In our ICU cohort, age, underlying pathologies, and mechanical ventilation did not differ between survivors and non-survivors, while SOFA score on admission was higher in non-survivors. Anti-COVID-19-directed treatment was similar in the two patient groups.

In our ICU patients, the vast majority of whom were mechanically ventilated, the mortality was 26%, indicating that many patients have survived COVID-19-related pathologies. Given the high mortality rates in these patients, however, early identification of prognostic indicators is crucial. Endothelial dysfunction has been shown to be directly involved in ARDS and sepsis [16], however its involvement in COVID-19 is being currently explored [17]. Markers of endothelial activation and dysfunction could be studied for increased risk of COVID-19-associated mortality. Hence, we hypothesized that the ICU admission levels of endothelial biomarkers in COVID-19 patients may predict mortality.

Plasma levels of VEGF, which stimulates the proliferation of vascular endothelial cells, have been associated with mortality in ICU septic patients [18,19]. Increased levels of soluble VE-cadherin, which controls the structure of intercellular junctions and endothelial cells, have also been associated with poor outcome in severe sepsis [20]. The high circulating levels found in septic patients has been suggested to reflect disruption of the endothelial barrier. Despite the role of these molecules in endothelial function and integrity, no differentiation was found between survivors and non-survivors in our study.

While Ang-1 maintains vessel integrity and inhibits vascular leakage, Ang-2 reflects vascular barrier breakdown [21,22]. Elevated ICU admission levels of Ang-2 have been associated with higher ICU sepsis-associated mortality [23,24]; Ang-2 was shown to be a relevant predictive factor for ICU direct admission in hospitalized COVID-19 patients, indicating that endothelial activation reinforces the hypothesis of a COVID-19-associated microvascular dysfunction [12]. Very recently, it was also demonstrated that Ang-2 was elevated in critically ill COVID-19 patients compared to controls and, moreover, it was strongly predictive of in-hospital mortality [25]. Our results agree with these findings; we also found elevated levels of Ang-2 in patients who will be admitted to the ICU as opposed to the ward, but most importantly, amongst the ICU patients, those who will eventually die in the ICU have even higher admission Ang-2 levels.

The migration of leukocytes across endothelial cells is an important step in the immune response [26]. ICAM-1, which controls the firm adhesion of neutrophils on the endothelium, has been extensively studied in relation to non-COVID-19 ICU outcome; ICAM-1 production has been shown to be associated with increased mortality [27,28]. In COVID-19, the association of sICAM-1 levels with patients’ severity was shown in ward patients, in whom serum levels of ICAM-1, amongst others, were dramatically elevated in severe cases, concluding that the increased expression of endothelial cell adhesion molecules is related to COVID-19 disease severity, and may contribute to coagulation dysfunction [29]. In our cohort, we found significantly higher sICAM-1 levels in those who eventually died in the ICU. Furthermore, we found elevated hospital admission levels in the patients admitted to the ICU compared to the ward. Thus, sICAM-1 might be used both as a predictive factor for ICU admission in hospitalized COVID-19 patients, and moreover, as a predictor of ICU mortality in such patients.

The selectin family, on the other hand, acts as a mediator of capture and rolling of leukocytes along the endothelium prior to their diapedesis at sites of tissue injury and inflammation [30]. Soluble levels of E-selectin, which is exclusively expressed on activated endothelial cells, are present at very low concentrations in healthy humans and are increased in various inflammatory conditions [31,32]. Hence, the soluble levels of E-selectin have been suggested to act as a circulating surrogate for the measurement of endothelial damage or activation [33]. A very recent study showed that COVID-19 patients admitted to the emergency department and directly transferred to the ICU had increased sE-selectin levels compared to patients who were admitted in conventional wards, without requiring ICU transfer during hospitalization [12]; in addition, another study showed that the sE-selectin levels were increased in patients with severe compared to mild disease [34]. The results of our study partly agree with these findings; we found a trend of elevated admission levels in the patients admitted to the ICU compared to those admitted to the ward, but we observed significantly higher levels in those who eventually died in the ICU. P-selectin has a similar function to E-selectin, however, it is constitutively expressed in lung endothelial cells and activated platelets, and correlates with lung endothelial injury and immunothrombosis [35]. Soluble P-selectin levels were shown to be significantly elevated in COVID-19 ICU patients compared to non-ICU patients [5]. In our cohort, we did not find higher levels of sP-selectin in critically ill COVID-19 patients compared to patients hospitalized in the ward; this is most possibly due to the fact that we assessed markers on hospital admission, rather than during hospitalization, as the study mentioned above. This could also explain the fact that we did not find elevated fibrinogen and D-dimers upon admission in ICU patients, suggesting that phenomena, such as platelet activation, as well as, dysregulation of the coagulation and fibrinolytic systems, may occur later than endothelial activation-dysfunction. It is of note that we did not find evidence of overt thromboembolic disease in any of our patients upon admission. However, we found elevated sP-selectin levels in ICU non-survivors compared to survivors, although sP-selectin did not show such a good discriminating ability from the generated ROC curve. In a similar respect, sP-selectin was found to be associated with thrombosis or death in patients hospitalized with COVID-19 [36].

Finally, vWF, an endothelial product that mediates platelet adhesion at sites of vascular damage, has been reported to be higher in patients with sepsis than those without [27]. In COVID-19, the plasma vWf levels have been shown to be markedly increased in patients with severe COVID-19 requiring ICU support [5,37,38,39]. We also found elevated admission levels in the patients admitted to the ICU compared to those admitted to the ward, and we observed significantly higher levels in those who will eventually die in the ICU.

It is worth noting that the principal molecules stored and rapidly released by exocytosis from Weibel–Palade bodies (i.e., the storage granules of endothelial cells), rendering them readily available, are vWf, Ang-2, and P-selectin [40]. All three were elevated in our non-survivors on ICU admission, implying early dysregulation of haemostasis and inflammation. Targeted inhibitors of exocytosis could probably contribute to the management of inflammatory or thrombotic conditions seen in COVID-19.

Our data add to the findings on various markers of endothelial cell and platelet activation, including vWf, soluble thrombomodulin, soluble CD40 ligand, and plasminogen activator inhibitor (PAI)-1, studied in COVID-19 [5,25,38]. Results from these reports have shown that these biomarkers were elevated in patients with critical COVID-19 infection.

In this study, we were able to demonstrate that in critically ill COVID-19 patients, circulating P-selectin, E-selectin, Ang-2, ICAM-1, and vWf levels on ICU admission are elevated in non-survivors compared to survivors. Interestingly, most biomarker levels were associated with each other. Furthermore, in our cohort the levels of these molecules, apart from vWf, acted as independent risk factors associated with ICU mortality with time. In addition, ROC curve analysis denoted that sE-selectin, Ang-2 and sICAM-1 had an equally good prognostic accuracy in identifying patients who will subsequently die in the ICU.

Our results also confirmed that ICU patients have higher levels of Ang-2, sE-selectin, and vWf compared with non-ICU hospitalized patients. We additionally demonstrated that sICAM-1 and vWf were elevated in non-survivors. The question remains whether the increased amounts of these biomarkers in plasma result from endothelial injury rather than mere activation. The ability of sE-selectin and sICAM-1 to identify patients who will subsequently die in the ICU may reflect the important role of both the endothelium and leukocytes in COVID-19, pinpointing that their interaction is of prominent significance.

The limitations of our study should be mentioned. Firstly, this was a single-center study, including a moderate number of patients, but comparable to similar studies. Regarding the statistical analysis, multivariate logistic regression analysis could not be performed in our study due to the limited number of patients. As a consequence, our simple scoring system was used based on the cut-off values obtained from the ROC curves to examine the cumulative effects of the biomarkers on mortality. A cross-center evaluation with a larger sample might provide more accurate results. Finally, none of our patients received dexamethasone, which is now part of the standard care, not allowing for analysis of the effect of corticosteroid treatments. Further mechanistic studies to understand the causes of endothelial injury, vascular dysfunction, and thrombosis are needed to provide vital insights into COVID-19 pathogenesis.

The clinical management of COVID-19 includes anticoagulation treatment, while immunomodulatory and anti-aggregation therapies are under investigation. Novel therapies for endotheliopathy, and vasculitis might be also needed. Developing tests to detect vascular injury may be critical to guide clinical management. The results from related studies might lead to the search for prognostic biomarkers, as well as therapeutics targeting pathogenic endothelial responses.

## 5. Conclusions

Studies until now have shown that elevated Ang-2 and sE-selectin levels in COVID-19 patients presenting to the emergency department are predictors for direct ICU admission. We have now further expanded this knowledge by demonstrating that increased Ang-2, sE-selectin, and sICAM-1 levels, apart from differentiating patients transferred to the ICU or the ward, are also associated with increased ICU mortality risk, probably reflecting the endothelial dysfunction that occurs in COVID-19. Rapidly emerging data on COVID-19 are providing insight into how endothelial dysfunction may contribute to the pandemic. This may lead to the search for prognostic biomarkers, as well as therapeutics targeting pathogenic endothelial responses. The results from related studies might help identify patients who require intensified management in an effort to achieve optimal triage and treatment.

## Figures and Tables

**Table 1:** Laboratory data of the critically ill patients upon intensive care unit (ICU) admission.

Characteristics	Survivors	Non–Survivors	*p*–Value	Reference Values
Number of patients, N	28	10		
Vitals signs				
Heart rate (bpm), (mean ± SD)	87 ± 17	102 ± 27	0.06	
Mean arterial pressure (mmHg), (mean ± SD)	81 ± 13	89 ± 23	0.2	
Respiratory rate (breaths/min), (mean ± SD)	21 ± 3	25 ± 4	0.004 *	
Temperature (°C), (mean ± SD)	37.5 ± 0.9	37.3 ± 1.4	0.6	
Laboratory data				
Hemoglobin, (mean ± SD)	13 ± 2	13 ± 2	0.3	12–17.5
Hematocrit, (mean ± SD)	40 ± 4	39 ± 5	0.8	37–51
White blood cell count (per μL), (mean ± SD)	9121 ± 3982	12972 ± 5380	0.02 *	4–10.5 × 10^3^
Neutrophlils (%), (mean ± SD)	79.8 ± 7.1	84.2 ± 5.8	0.1	40–70
Lymphocytes (%), (mean ± SD)	13.9 ± 6.1	10.4 ± 4.6	0.1	25–45
Platelets (per μL), (median, IQR)	207,000 (144,000–258,000)	267,000 (194,500–394,750)	0.1	140–450 × 10^3^
PT (s), (median, IQR)	13 (12–14)	14 (13–14)	0.9	11.0–12.5
APTT (s), (mean ± SD)	35 ± 6	35 ± 5	0.7	26–38
INR, (median, IQR)	1.08 (1.01–1.11)	1.11 (1.04–1.17)	0.3	0.8–1.1
Creatinine (mg/dL), (mean ± SD)	0.94 ± 0.28	1.21 ± 0.39	0.03 *	0.6–1.4
Glucose (mg/dL), (median, IQR)	124 (109–189)	178 (137–203)	0.3	70–110
Total bilirubin (mg/dL), (median, IQR)	0.7 (0.4–0.8)	0.6 (0.5–1.2)	0.5	<1
Total protein (g/dL), (mean ± SD)	6.3 ± 0.6	6.0 ± 1.0	0.2	6–8.2
Albumin (g/dL), (mean ± SD)	3.5 ± 0.6	3.2 ± 0.6	0.1	3.5–5.0
Globulin (g/dL), (mean ± SD)	2.7 ± 0.4	2.8 ± 0.7	0.8	2.3–3.5
CKMB (IU/), (median, IQR)	19 (16–37)	24 (21–37)	0.3	1–18
CK (U/L), (median, IQR)	166 (71–321)	122 (67–575)	0.8	10–173
Fibrinogen (mg/dL), (mean ± SD)	635 ± 165	626 ± 174	0.9	200–400
D–dimers (µg/mL), (median, IQR)	0.33 (0.20–0.59)	0.52 (0.34–1.0)	0.3	<0.5
CRP (mg/dL), (median, IQR)	11.4 (5.9–19.0)	13.5 (4.9–28.2)	0.5	<0.5
γ–GT (IU/L), (median, IQR)	47 (22–79)	74 (26–175)	0.2	7–49
Urea (mg/dL), (median, IQR)	31 (25–55)	49 (30–93)	0.1	10–50
AST (IU/L), (median, IQR)	42 (36–59)	55 (33–70)	0.3	5–37
ALT (IU/L), (median, IQR)	39 (23–56)	48 (25–78)	0.3	5–40
Na^+^ (mEq/L), (mean ± SD)	138 ± 5	142 ± 7	0.04 *	135–147
K^+^ (mEq/L), (mean ± SD)	4.2 ± 0.5	4.4 ± 0.6	0.2	3.5–5.1
ALP (U/L), (median, IQR)	56 (43–76)	91 (66–183)	0.1	35–129
LDH (U/L), (median, IQR)	418 (335–629)	533 (387–642)	0.9	<225
Troponin (ng/mL), (median, IQR)	12 (10–27)	63 (13–133)	0.2	<14
Amylase (U/L), (median, IQR)	66 (53–114)	66 (36–111)	0.5	10–100
Lactate (mmol/L), (mean ± SD)	1.2 ± 0.4	1.2 ± 0.5	0.6	<2.0

* *p*-value < 0.05. Data are expressed as the number of patients (N), percentages of total related variable (%), and mean ± SD for normally distributed variables and median (IQR) for skewed data. Patients were divided in 2 groups depending on ICU mortality. For differences between the 2 groups, either the Student’s t-test for normally distributed data or the Mann–Whitney test for skewed data was used. Vital signs listed are the most abnormal recorded during the 24-h post ICU admission, while laboratory data were measured once (within 24 h from ICU admission). Definition of abbreviations: γ-GT = γ-Glutamyl transpeptidase; ALP = Alkaline phosphatase; ALT = Alanine transaminase; APTT = Activated partial thromboplastin time; AST = Aspartate transaminase; CK = Creatine kinase; CKMB = Creatinine kinase Myocardial band; CRP = C-reactive protein; ICU = Intensive care unit; INR = International normalized ratio; LDH = Lactate dehydrogenase; PT = Prothrombin time.

**Table 2:** Demographics, clinical characteristics, and endothelial biomarkers of the critically ill patients upon intensive care unit (ICU) admission.

Characteristics	Survivors	Non-Survivors	*p*-Value
Number of patients, N	28	10	
Age (years), (mean ± SD)	62 ± 11	68 ± 10	0.2
Sex, N (%)			0.9
Male	23 (82.1%)	8 (80.0%)	
Female	5 (17.9%)	2 (20.0%)	
Comorbidities, N (%)	18 (64.3%)	7 (70.0%)	0.7
Hypertension	12	5	
Diabetes	3	2	
CAD	3	1	
COPD	1	0	
Asthma	0	1	
Hyperlipidemia	7	0	
Hepatitis	0	1	
Sick days prior to ICU admission (mean ± SD)	6 ± 3	7 ± 2	0.8
Characteristics on ICU admission			
APACHE II, (mean ± SD) SOFA, (mean ± SD) PaO2/FiO2 (mmHg), (mean ± SD) PCO2 (mmHg), (median, IQR) pH, (mean ± SD) HCO3 (mEq/L), (mean ± SD)	14 ± 5 6 ± 3 202 ± 90 41 (33–47) 7.4 ± 0.1 25 ± 4	16 ± 4 9 ± 2 175 ± 71 48 (43–56) 7.3 ± 0.1 25 ± 5	0.2 0.02 * 0.5 0.06 0.04 * > 0.9
Endothelial markers			
sE-selectin (ng/mL), (median, IQR) sP-selectin (ng/mL), (median, IQR) Angiopoietin-1 (ng/mL), (median, IQR) Angiopoietin-2 (ng/mL), (median, IQR) Ang-2:Ang-1 (median, IQR) VEGF (ng/mL), (median, IQR) VE-cadherin (ng/mL), (median, IQR) sICAM (ng/mL), (median, IQR) vWf (ng/mL), (median, IQR)	33.7 (25.0–45.5) 89.2 (52.4–161.2) 7.3 (3.2–22.4) 2.4 (1.5–4.3) 0.17 (0.04–0.35) 144.1 (118.2–269.6) 1337 (1005–1833) 211.2 (159.7–388.3) 8.52 (6.74–13.36)	60.6 (49.2–111.6) 152.7 (122.1–223.7) 5.2 (1.5–22.4) 10.7 (5.4–15.2) 1.18 (0.18–2.80) 85.5 (45.9–265.8) 1238 (1125–2247) 614.5 (309.7–781.8) 15.76 (9.92–33.70)	<0.001 * 0.03 * 0.6 0.001 * 0.1 0.3 0.7 <0.001 * 0.008 *
COVID-19-targeted treatment	28 (100.0%)	10 (100.0%)	
Azithromycin/chloroquine/lopinavir/ritonavir Azithromycin/chloroquine Lopinavir/ritonavir/chloroquine Chloroquine Azithromycin Convalescent Plasma Other	6 (21.4%) 9 (32.1%) 1 (3.6%) 2 (7.2%) 4 (14.3%) 3 (10.7%) 3 (10.7%)	5 (50.0%) 2 (20.0%) 1 (10.0%) 0 (0.0%) 0 (0.0%) 0 (0.0%) 2 (20.0%)	
Outcomes			
LoS in the ICU (days), (median, IQR) Mechanical ventilation, N (%) Duration of mechanical ventilation (days), (median, IQR)	14 (11–30) 20 (71.4%) 12 (4–35)	29 (17–40) 10 (100.0%) 26 (17–40)	0.5 0.06 0.4

* *p*-value < 0.05. Data are expressed as the number of patients (N), percentages of total related variable (%), and mean ± SD for normally distributed variables and median (IQR) for skewed data. Patients were divided in 2 groups depending on ICU mortality. For differences between the 2 groups, either the Student’s *t*-test for normally distributed data, the Mann–Whitney test for skewed data, or the chi-square test for nominal data was used. Characteristics were measured once (within 24 h from ICU admission). Definition of abbreviations: Ang = Angiopoietin; APACHE = Acute physiology and chronic health evaluation; CAD = Coronary artery disease; COPD = Chronic obstructive pulmonary disease; ICU = Intensive care unit; LoS = Length of stay; sICAM-1 = soluble Intercellular adhesion molecule 1; SOFA = Sequential organ failure assessment; VE-cadherin= Vascular endothelial cadherin; VEGF = Vascular endothelial growth factor; vWf = von Willebrand factor.

## Data Availability

Available upon reasonable request.

## Supplementary Materials

The following are available online at https://www.mdpi.com/2073-4409/10/1/186/s1: Table S1: Laboratory data on hospital admission of ICU and non-ICU patients; Table S2: Demographics, clinical characteristics, and endothelial biomarkers on hospital admission of ICU and non-ICU patients.
