# Supplementary material for: ICU Admission Levels of Endothelial Biomarkers as Predictors of Mortality in Critically Ill COVID-19 Patients
Source: Cells. 2021 Jan 19;10(1):186. doi: 10.3390/cells10010186 (PMC7832393; doi:10.3390/cells10010186)
Supplement: Supplementary file 1 [file cells-10-00186-s001.zip › Table S1_Cells.docx]

**Table S1.** Laboratory data on hospital admission of ICU and non-ICU patients.

| **Characteristics** | **ICU** | **Non-ICU** | ***p*-Value** |
| --- | --- | --- | --- |
| Number of patients, N  Vitals signs  Heart rate (bpm), (median, IQR)  Mean arterial pressure (mmHg), (median, IQR)  Respiratory rate (breaths/min), (mean ± SD)  Temperature (^o^C), (median, IQR)  Laboratory data  Hemoglobin, (median, IQR)  Hematocrit, (median, IQR)  White blood cell count (per μL), (mean ± SD)  Neutrophils (%), (mean ± SD)  Lymphocytes (%), (mean ± SD)  Platelets (per μL), (median, IQR)  PT (sec), (median, IQR)  APTT (sec), (mean ± SD)  INR, (median, IQR)  Creatinine (mg/dL), (median, IQR)  Glucose (mg/dL), (median, IQR)  Total bilirubin (mg/dL), (median, IQR)  Total protein (g/dL), (mean ± SD)  Albumin (g/dL), (mean ± SD)  Globulin (g/dL), (mean ± SD)  CKMB (IU/L), (median, IQR)  CK (U/L), (median, IQR)  Fibrinogen (mg/dL), (mean ± SD)  D-dimers (µg/mL), (median, IQR)  CRP (mg/dL), (median, IQR)  γ-GT (IU/L), (median, IQR)  Urea (mg/dL), (median, IQR)  AST (IU/L), (median, IQR)  ALT (IU/L), (median, IQR)  Na^+^ (mEq/L), (mean ± SD)  K^+^ (mEq/L), (mean ± SD)  ALP (U/L), (median, IQR)  LDH (U/L), (median, IQR)  Troponin (ng/mL), (median, IQR)  Amylase (U/L), (median, IQR)  Lactate (mmol/L), (mean ± SD) | 38  87 (81-105)  78 (72-89)  22 ± 4  37.7 (37.2-38.0)  14 (12-14)  40 (38-42)  10162 ± 46.58  81.0 ± 6.9  12.9 ± 5.9  219000 (163000-269500)  13 (13-14)  35.1 ± 5.6  1.08 (1.00-1.12)  0.9 (0.8-1.2)  147 (112-188)  0.64 (0.49-0.84)  6.2 ± 0.7  3.4 ± 0.6  2.7 ± 0.5  23.0 (17.0-37.0)  137.0 (70.0-337.5)  632.6 ± 165.1  0.46 (0.20-0.60)  11.5 (5.3-19.6)  59.0 (23.0-100.0)  38.0 (27.0-55.5)  43 (35-62)  41 (25-57)  138.6 ± 5.3  4.3 ± 0.6  67 (48-114)  425 (344-632)  14 (10-50)  66 (46-114)  1.2 ± 0.4 | 17  90 (82-90)  77 (70-88)  21 ± 4  38.4 (37.0-28.8)  13 (12-14)  39 (34-40)  7761 ± 4190  72.9 ± 13.7  19.7 ± 11.2  204500 (165250-273000)  13 (12-14)  31.2 ± 5.3  1.06 (0.99-1.16)  0.9 (0.7-1.1)  111 (97-133)  0.40 (0.26-0.78)  6.5 ± 0.5  3.7 ± 0.4  2.8 ± 0.4  23.0 (16.5-28.0)  97.5 (66.3-201.8)  567.5 ± 205.5  0.74 (0.33-1.06)  6.4 (2.6-15.4)  31.5 (16.3-61.5)  29.5 (18.5-38.0)  27 (19-52)  23 (17-46)  134.9 ± 5.3  4.3 ± 0.7  65 (51-87)  280 (223-411)  10 (3-20)  62 (55-73)  1.1 ± 0.3 | 0.8  0.3  0.5  0.2  0.5  0.7  0.008*  0.006*  0.005*  0.08  0.3  0.02*  0.3  0.5  0.9  0.04*  0.2  0.1  0.8  0.2  0.08  0.3  0.2  0.5  0.045*  0.2  0.02  0.2  0.02*  > 0.9  0.1  0.006*  0.1  0.2  0.5 |

*p-value< 0.05. Data are expressed as number of patients (N), percentages of total related variable (%) and mean ± SD for normally distributed variables and median (IQR) for skewed data. For differences between the 2 groups, either the Student’s t-test for normally distributed data or the Mann-Whitney test for skewed data was used. Vital signs listed are the most abnormal recorded during the 24-hours post-admission, while laboratory data were measured once (within 24-hours from admission). For the reference values, please see Table 1. Definition of abbreviations: γ-GT= γ-Glutamyl transpeptidase; ALP= Alkaline phosphatase; ALT= Alanine transaminase; APTT= Activated partial thromboplastin time; AST= Aspartate transaminase; CK= Creatine kinase; CKMB= Creatinine kinase myocardial band; CRP= C-reactive protein; ICU= Intensive care unit; INR= International normalized ratio; LDH= Lactate dehydrogenase; PT= Prothrombin time.
