# Supplementary material for: ICU Admission Levels of Endothelial Biomarkers as Predictors of Mortality in Critically Ill COVID-19 Patients
Source: Cells. 2021 Jan 19;10(1):186. doi: 10.3390/cells10010186 (PMC7832393; doi:10.3390/cells10010186)
Supplement: Supplementary file 1 [file cells-10-00186-s001.zip › Table S2_Cells.docx]

**Table S2.** Demographics, clinical characteristics, and endothelial biomarkers on hospital admission of ICU and non-ICU patients.

| **Characteristics** | **ICU** | **Non-ICU** | ***p*-Value** |
| --- | --- | --- | --- |
| Number of patients, N (%) | 38 | 17 |  |
| Age (years), (mean ± SD) | 63 ± 11 | 55 ± 17 | 0.03* |
| Sex, N (%) |  |  | 0.5 |
| Male | 31 (81.6%) | 12 (70.6%) |  |
| Female | 7 (18.4%) | 5 (29.4%) |  |
| Comorbidities, N (%)  Hypertension  Diabetes  CAD  COPD  Asthma  Hyperlipidemia  Hepatitis | 25 (65.8%)  17  5  4  1  1  9  1 | 8 (47.1%)  6  2  3  0  0  3  0 | 0.2 |
| Sickness days prior to admission (mean ± SD)  Characteristics on admission | 6 ± 2 | 9 ± 6 | 0.01* |
| SOFA, (mean ± SD) | 7 ± 3 | 2 ± 1 | < 0.0001* |
| PaO_2_/FiO_2_ (mmHg), (mean ± SD)  PCO_2_ (mmHg), (mean ± SD)  pH, (mean ± SD)  HCO_3_ (mEq/L), (mean ± SD) | 195 ± 85  45 ± 12  7.4 ± 0.1  25 ± 4 | 302 ± 93  33 ± 4  7.4 ± 0.5  23 ± 3 | < 0.0001*  < 0.0001*  0.04*  0.3 |
| Endothelial markers  sE-selectin (ng/mL), (median, IQR)  sP-selectin (ng/mL), (median, IQR)  Angiopoietin-1 (ng/mL), (median, IQR)  Angiopoietin-2 (ng/mL), (median, IQR)  Ang-2:Ang-1 (median, IQR)  VEGF (ng/mL), (median, IQR)  VE-cadherin (ng/mL), (median, IQR)  sICAM-1 (ng/mL), (median, IQR)  vWf (ng/mL), (median, IQR)  Outcomes  LoS (days), (median, IQR)  Mortality, N (%) | 43.01 (28.05-54.99)  112.7 (63.97-172.7)  6.86 (2.71-21.13)  3.51 (1.54-10.10)  0.28 (0.14-1.89)  142.8 (85.5- 265.2)  1275 (1044-1844)  263.2 (170.6-537.5)  10.08 (7.76-16.08)  18 (12-38)  10 (26.3%) | 25.35 (16.14-46.99)  140.5 (87.99-236.5)  13.58 (9.38-23.42)  1.83 (0.99-3.47)  0.12 (0.05-0.25)  267.5 (121.5-487.8)  1415 (1097-1771)  168.1 (128.1-218.7)  5.38 (4.02-9.01)  12 (8-16)  0 (0.0%) | 0.06  0.3  0.2  0.01*  0.008*  0.08  0.7  0.008*  0.001*  0.001*  0.02* |

*p-value< 0.05. Data are expressed as number of patients (N), percentages of total related variable (%) and mean ± SD for normally distributed variables and median (IQR) for skewed data. For differences between the 2 groups, either the Student’s t-test for normally distributed data, the Mann-Whitney test for skewed data, or the chi-square test for nominal data was used. Characteristics were measured on hospital admission (within 24 hours). Definition of abbreviations: Ang= Angiopoietin; CAD= Coronary artery disease; COPD= Chronic obstructive pulmonary disease; ICU= Intensive care unit; LoS= Length of stay in ICU or the ward; sICAM-1= soluble Intercellular adhesion molecule 1; VE-cadherin= Vascular endothelial cadherin; VEGF= Vascular endothelial growth factor; vWf= von Willebrand factor.
